# Antimicrobial, Cytotoxic and Mutagenic Activity of Gemini QAS Derivatives of 1,4:3,6-Dianhydro-l-iditol

**DOI:** 10.3390/molecules27030757

**Published:** 2022-01-24

**Authors:** Karol Sikora, Andrzej Nowacki, Piotr Szweda, Anna Woziwodzka, Sylwia Bartoszewska, Jacek Piosik, Barbara Dmochowska

**Affiliations:** 1 Department of Inorganic Chemistry, Faculty of Pharmacy, Medical University of Gdańsk, Al. Gen. J. Hallera 107, 80-416 Gdańsk, Poland; sylwia.bartoszewska@gumed.edu.pl; 2 Faculty of Chemistry, University of Gdańsk, Wita Stwosza 63, 80-308 Gdańsk, Poland; andrzej.nowacki@ug.edu.pl (A.N.); basia.dmochowska@ug.edu.pl (B.D.); 3 Department of Pharmaceutical Technology and Biochemistry, Faculty of Chemistry, Gdańsk University of Technology, ul. G. Narutowicza 11/12, 80-233 Gdańsk, Poland; piotr.szweda@pg.edu.pl; 4 Laboratory of Biophysics, Intercollegiate Faculty of Biotechnology, University of Gdańsk and Medical University of Gdańsk, Abrahama 58, 80-307 Gdańsk, Poland; anna.woziwodzka@biotech.ug.edu.pl (A.W.); jacek.piosik@biotech.ug.edu.pl (J.P.)

**Keywords:** quaternary ammonium salts, gemini, 1,4:3,6-dianhydro-d-mannitol, antimicrobial activity, mutagenicity

## Abstract

A series of quaternary diammonium salts derivatives of 1,4:3,6-dianhydro-l-iditol were synthesized, using isommanide (1,4:3,6-dianhydro-d-mannitol) as a starting material. Both aromatic (pyridine, 4-(*N*,*N*-dimethylamino)pyridine (DMAP), (3-carboxamide)pyridine; *N*-methylimidazole) and aliphatic (trimethylamine, *N*,*N*-dimethylhexylamine, *N*,*N*-dimethyloctylamine, *N*,*N*-dimethyldecylamine) amines were used, giving eight gemini quaternary ammonium salts (QAS). All salts were tested for their antimicrobial activity against yeasts, *Candida albicans* and *Candida glabrata*, as well as bacterial *Staphylococcus aureus* and *Escherichia coli* reference strains. Moreover, antibacterial activity against 20 isolates of *S. aureus* collected from patients with skin and soft tissue infections (*n* = 8) and strains derived from subclinical bovine mastitis milk samples (*n* = 12) were evaluated. Two QAS with octyl and decyl residues exhibited antimicrobial activity, whereas those with two decyl residues proved to be the most active against the tested pathogens, with MIC of 16–32, 32, and 8 µg/mL for yeast, *E. coli,* and *S. aureus* reference and clinical strains, respectively. Only QAS with decyl residues proved to be cytotoxic in MTT assay against human keratinocytes (HaCaT), IC_50_ 12.8 ± 1.2 μg/mL. Ames test was used to assess the mutagenic potential of QAS, and none of them showed mutagenic activity in the concentration range 4–2000 µg/plate.

## 1. Introduction

Rising consumption, depleting fossils fuels, and environmental challenges are motivating chemists, biologists, and biotechnologists to search for new chemicals derived from renewable resources. Except for new compounds with superior properties, a search for alternative ways of obtaining crucial chemicals is an important issue. The most prominent renewable resource is biomass; it can be used both as a source of energy and a wide variety of chemicals. The main advantage of biomass in the production of energy and chemicals is that it can be obtained as a byproduct of agricultural and forest industries. Among others, sugars, alditols, and anhydroalditols can easily be generated in large quantities by biomass processing, making it a convenient platform for the synthesis of new active compounds [1,2,3]. Recently, it was established that sugars are not only the energy source for living organisms. They are important building blocks of many organisms forming cellulose, chitin, DNA, and RNA. Moreover, some of them are antibiotics (i.e., gentamycin) [4] and play a crucial role in cell–cell interactions [5]. Sugars are recognized by proteins to induce multiple processes, such as cell-cell recognition/adhesion, the interaction of cells with the external environment, infection of pathogens, and immune response. Moreover, glycocalyx, the sugar outer layer of cells, protects them from external conditions and also enables red blood cells to pass through capillary vessels [6,7].

Quaternary ammonium salts (QAS) are a class of cationic surfactants consisting of a positively charged nitrogen atom, and they have multiple applications in almost every area of modern society. In medicine, QAS are used as antimicrobial agents in disinfectants and antiseptics, as preservatives in drugs, as muscle relaxants in anesthesia and pulmonary disorders [8,9,10,11]. They are also effective against G+ and G− bacteria, fungi, and parasites. A special case of QASs are diammonium salts, which are commonly called gemini salts. Gemini salts exhibit an even stronger biological activity than that of monoammonium ones. Active concentrations of diammonium salts are even 1000 times lower than those of their monoammonium analogues [8,12]. Gemini QAS are also investigated for application in transfection as an alternative for viral vectors for introducing DNA or RNA to cells [13]. The highest annual consumption of QAS was recorded in households (fabric softeners, shampoos) and agriculture (pesticides and herbicides) [14,15,16]. Furthermore, QAS are used in many branches of industry and technology as catalysts in phase transfer catalysis (PTC), corrosion inhibitors and solvents with low vapor pressure, and as ionic liquids (IL) [16,17]. Properties of QAS made them useful in the laboratory, including asymmetric synthesis, chiral resolution, and as shift reagents in NMR [18,19,20]. Despite their usefulness, QAS have some limitations, such as limited biodegradation [21,22,23], risk of pollution of the environment [24,25,26], development of bacterial resistance and the risk of mutagenicity [27]. Commonly accepted mechanism of antimicrobial activity assumes interaction of QAS with the cell membrane. In the first stage, positively charged molecules are attracted to negatively charged phospholipids of the cell membrane. Next, the hydrophobic tail of the molecule penetrates to the inner part of bilayer membrane and disrupts its structure. The consequence of that is the loss of structural integrity of the cell, leak of low molecular components and finally autolysis. Despite numerus studies, the mechanism of antimicrobial activity of QAS is not fully understood [8,28].

Combining the properties of QAS and sugars creates a class of compounds that are a promising alternative for currently used drugs and chemicals. This approach fits perfectly with the green chemistry approach for the search for new active compounds. Despite their potential applications, the body of literature on QAS with sugar/alditol moieties is rather poor as compared to that of non-sugar based QAS. Recently, carbohydrate-based QAS gained more attention [29,30,31,32]. For example, a series of QAS derivatives of alditols, cyclodextrins, cellulose and carbohydrates were synthesized by Engel et al. [33,34,35,36]. They synthesized in particular 1,4-diazabicyclo[2.2.2]octane (DABCO) derivatives linked either to C6 carbohydrate carbon atom or terminal carbon of a linear alditol. The use of DABCO opens the way for a straightforward modification of hydrophobicity of QAS by attachment of alkyl substituents with different chain lengths to one of the nitrogen atoms of DABCO. Some of those new compounds were tested for activity against *Staphylococcus aureus* strains. Mannitol derivatives carrying two DABCO units modified with 16 C and 18 C hydrocarbons exhibited excellent antistaphylococcal activity at minimum inhibitory concentrations (MIC) of 9.1 and 2.2 μM, respectively [34]. Moreover, the modified cellulose containing DABCO with a hexyldecyl residue inhibited the growth of both G+ and G− bacteria on the modified material [35]. An extensively explored area of research on modified sugar derivatives was offered by chitosan. This d-glucosamine polymer has an amine group attached to the C2 carbon atom of the sugar moiety and can be modified by a variety of substituents including quaternization. Some attempts were made to modify this material to apply it in medicine, i.e., for antimicrobial wound dressings or CO_2_ capture [37,38,39].

In recent years, 1,4:3,6-dianhydrohexitols have gained significant interest, apparently because of their strong potential in the synthesis of diversely functionalized compounds and availability from natural sources. These compounds can also be obtained in the laboratory in a quite simple procedure and be successfully used for the QAS synthesis. Mono- and diammonium derivatives of isosorbide (1,4:3,6-dianhydro-d-glucitol) and isomannide (1,4:3,6-dianhydro-d-mannitol) were reported. A series of isommanide derivatives with *N*-alkylated imidazole were synthesized by da Silva et al. [40]. Moreover, mono- and diammonium derivatives of isomannide with a *N,N*-dimethylbenzylamine moiety were synthesized and investigated for chiral resolution in NMR [41,42]. Furthermore, a series of isosorbide QASs derivatives of benzyl and dibenzylamine and their application for PTC were reported by Kumar et al. [43]. These compounds enhanced stereoselectivity in the benzylation reaction of *N*-(diphenylmethylene)glycine *tert*-butyl ester. The *S/R* isomers ratio after synthesis depended on both the position of the nitrogen atom and the configuration of the 1,4:3,6-dianhydrohexitol residue.

This work is the continuation of our studies on the formation, structural and mechanistic considerations as well as comprehensive biological activity of QAS. Previously, we described the synthesis and structural analysis of a series of QAS derivatives of 1,4:3,6-dianhydro-d-mannitol [44]. Here, we present the synthesis of a family of gemini QAS derived from 1,4:3,6-dianhydro-d-mannitol and analysis of their biological activity. This study aimed to investigate the antimicrobial, cytotoxic, and mutagenic activity of gemini QAS consisting of rigid 1,4:3,6-dianhydrohexitol scaffold and aliphatic and aromatic amine moieties Importantly, 1,4:3,6-dianhydro-d-mannitol can be obtained in large quantities from natural sources as a byproduct of the agriculture industry, and it is biodegradable and environmentally friendly. Moreover, the configuration of asymmetric centers allows efficient synthesis of QAS according to the S_N_2 mechanism. Aromatic (pyridine, DMAP, (3-carboxamide)pyridine, *N*-methylimidazole) and tertiary aliphatic amines with different chain lengths (hexyl, octyl, decyl) were used to evaluate the effect of type of residues attached to a quaternary nitrogen atom on biological activity.

The main idea behind these studies is to design and synthesize QAS that possess many valuable properties, such as high biological activity, nontoxicity, and biodegradability. We assume that such features could make these new compounds the successors of the currently used QAS, which has numerous properties unfavorable to the environment and health of humans and other organisms.

## 2. Results and Discussion

### 2.1. Chemistry

**Scheme 1:**
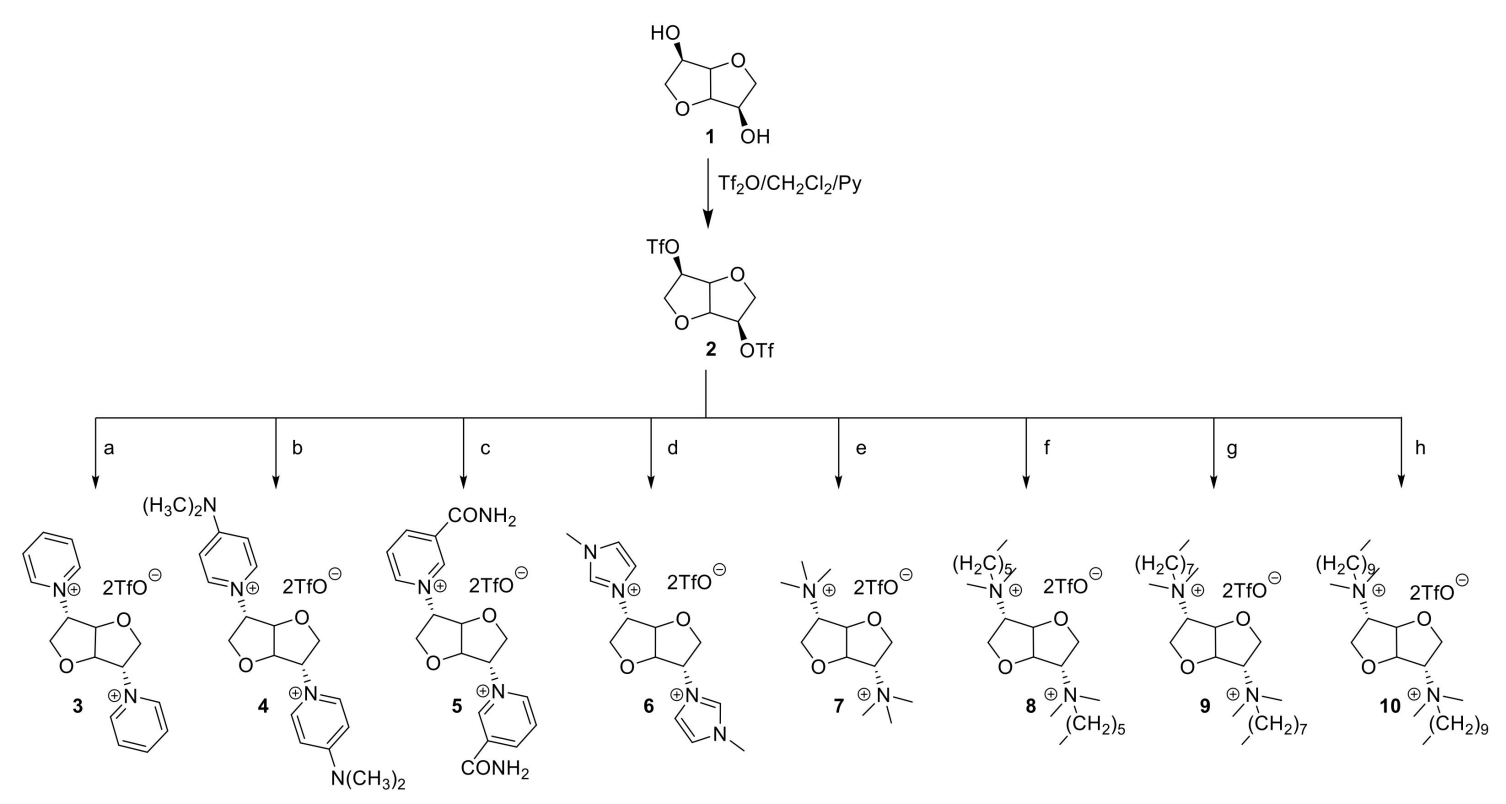
Synthesis of gemini QAS derivatives of 1,4:3,6-dianhydro-l-iditol. Reaction conditions: (**a**) pyridine, 48 h, RT; (**b**) DMAP, CH_3_CN, 48 h 40 °C; (**c**) pyridinie-3-carboxamide, CH_3_CN, 7 days, 70 °C; (**d**) *N*-methylimidazole, 24 h, RT; (**e**) trimethylamine in ethanol, 48 h, 40 °C; (**f**) *N,N*-dimethylhexylamine, CH_3_CN, 24 h, 70 °C; (**g**) *N,N*-dimethyloctylamine, CH_3_CN, 24 h, 70 °C; (**h**): *N,N*-dimethyldecylamine, CH_3_CN, 24 h, 70 °C.

All gemini quaternary ammonium salts were prepared in a two-step synthesis. First, commercial 1,4:3,6-dianhydro-d-mannitol (**1**) was treated with trifluoromethanosulfonic anhydride in the presence of pyridine in dry dichloromethane to yield 1,4:3,6-dianhydro-2,5-di-*O*-trifluoromethanesulfonyl-d-mannitol (**2**). Next, in the reaction with aliphatic and aromatic amines compound **2** provided a series of quaternary diammonium triflates **3**–**10** in excellent yields. The syntheses of pyridinium **3**, dimethylaminopyridinium **4**, trimethylammonium **7** and *N*,*N*-dimethyloctylammonium **9** derivatives were performed as previously reported [44]. The remaining QAS derivatives of aliphatic amines, *N*,*N*-dimethylhexylamine **8** and *N,N*-dimethyldecylamine **10** were prepared analogically to QAS **9**. The syntheses of **5** (a derivative of (3-carboxamide)pyridine) and **6** (a derivative of *N*-methylimidazole) were performed under modified conditions (Scheme 1). For the synthesis of **5**, a mixture of **2** and (3-carboxamide)pyridine in acetonitrile was kept in the heating block at 70 °C for 5 days. Then, the product was purified using FLASH chromatography. In contrary to other QAS synthesized, a lower yield (56%) was obtained. This can be explained as being due to the side-reactions of (3-carboxamide)pyridine, such as hydrolysis of the amide to acid and/or decarboxylation [45]. Since *N*-methylimidazole is liquid at ambient temperature and is an excellent nucleophile, as compared to other aromatic amines, used in this study, the synthesis of **6** was performed without any solvent, at room temperature for 24 h. After purification by extraction, the product was obtained in a high (96%) yield.

**Figure 1:**
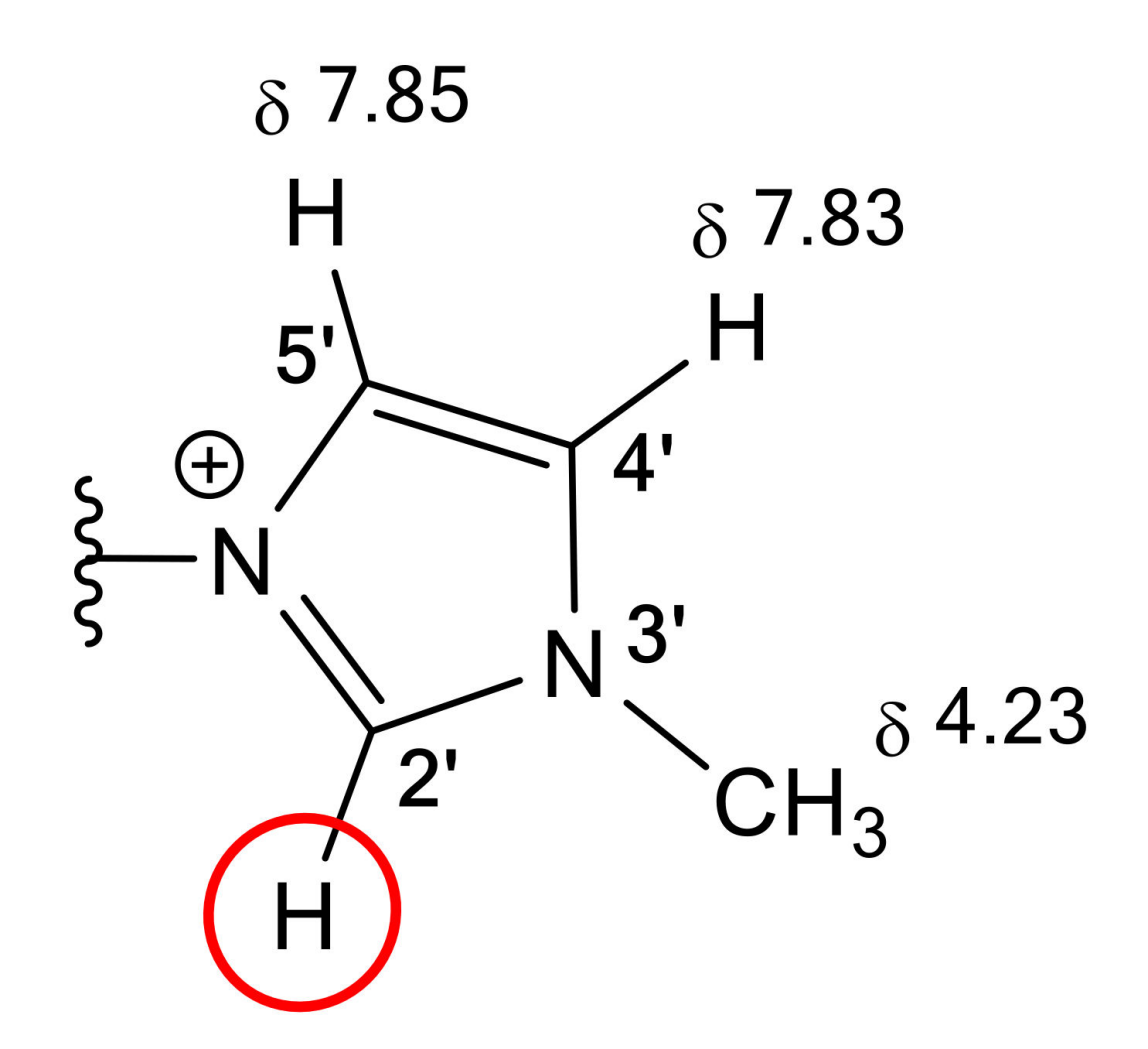
Structure and proton chemical shifts in ^1^H NMR spectra of *N*-methylimidazole moiety of QAS **6**.

All compounds were characterized using high-resolution mass spectrometry (HRMS) and nuclear magnetic resonance (^1^H, ^13^C, COSY and HSQC). The configuration inversion within the dianhydrohexitol moiety from d-manno to l-ido was noticed during the synthesis of QAS. The Walden inversion accompanied by the S_N_2 mechanism of the reaction was proven by analysis of the ^1^H NMR spectra. Moreover, HRMS analysis confirmed the molecular masses of the compounds. For all QAS, molecular ions with *m*/*z* corresponding to half of the monoisotopic masses of cations ([M]^2+^) was found. Because of the symmetry and magnetic equivalence of nuclei of the 1,4:3,6-dianhydrohexitol moiety *C_2_*, a reduction in the number of ^1^H NMR signals was seen. Moreover, the 2′ proton signal was missing in ^1^H NMR spectrum of compound **6** carrying the *N*-methylimidazole group. This phenomenon was caused by the acidity of a proton attached to a 2′ carbon atom of the imidazole ring and its exchange in the deuterated solvent, D_2_O, used for NMR (Figure 1) [46]. All results confirmed the structure of the compounds.

### 2.2. Antimicrobial Activity

The synthesized gemini QAS was tested for antimicrobial activity using the microdilution method. Two reference yeast strains, *Candida albicans* ATCC 10231 and *Candida glabrata* DSMZ 11226; and two reference bacterial strains, *Staphylococcus aureus* and *Escherichia coli* were used for preliminary assessment of the antimicrobial potential of all synthesized agents. The results are summarized in Table 1. QAS containing pyridinium **3**, dimethylaminopyridinium **4**, (3-carboxamide)pyridinium **5** *N*-methylimidazolium **6**, trimethylammonium **7** and *N,N*-dimethylhexylammonium **8** groups did not show any antimicrobial activity against the tested strains both bacterial and yeast with MIC value > 4000 μg/mL. But compound **9**, carrying two octyl groups in its structure, showed a weak activity against the tested yeast (MIC 1024 μg/mL) and *E. coli* (MIC 2048 μg/mL) and a higher activity against *S. aureus* with MIC 128 μg/mL. QAS **10** with two decyl hydrocarbon chains showed excellent activity of all synthesized salts with MIC values of 32 (*C. albicans*), 16 (*C. glabrata*), 8 (*S. aureus*), and 32 μg/mL (*E. coli*). Benzalkonium chloride (BAC) is a QAS commonly used as a preservative in drug formulations and as an active ingredient of disinfectants. BAC has higher antimicrobial activity than QAS **10**, i.e., MIC 8 μg/mL against *C. albicans* [47], 13 and 2 μg/mL against *E. coli* and *S. aureus,* respectively [48,49]. The antimicrobial activity of QAS mainly depends on the balance between the hydrophobic and hydrophilic parts of the molecule. For QAS **3**–**8** that do not possess hydrophobic reside no antimicrobial activity was observed. However, modification of residues attached to aromatic imidazolium rings can result in more potent antimicrobial compounds. The results indicate that elongation of the hydrocarbon chain intensifies antimicrobial activity in the order hexyl < octyl < decyl. This finding is consistent with that of the literature [8]. For monomeric QAS, the optimum length of the hydrophobic residue was established for 10 to 12 carbon atoms. With shorter chains, the hydrophobic part of the molecule was unable to interact with membrane phospholipids and disrupt their structure, while an increase in the chain length causes suppressed solubility of the surfactants in water. A more complex situation was noticed for gemini salts, where the hydrophobic/hydrophilic balance depends not only on hydrocarbon chain length and substituents of ammonium atom. With gemini salts, the type of spacer, its rigidity and the tendency toward conformational changes as well as between hydrophobic and hydrophilic parts have to be considered [8]. The suppressed water solubility of gemini salts **9** and **10** was noticed, and DMSO had to be used for solubilisation during antimicrobial assays. Furthermore, two compounds: **9** and **10** were tested for antistaphylococcal activity against clinical isolates collected from patients with skin and soft tissue infections as well as strains derived from subclinical bovine mastitis milk samples infections. In this study, 20 strains were used, and MIC values are summarized in Table 2. In general, the activity of salt **9** was higher by one-fold in comparison to that of the reference *S. aureus* strain (MIC 128 vs. 64 μg/mL). For **10**, with decyl hydrocarbon chains, similar results for clinical isolates and reference strains were seen (MIC 8 μg/mL). More susceptible strains with MIC 4 μg/mL could also be recorded. Only minor differences in the susceptibility of MRSA and MSSA isolates were found.

All synthesized QAS and benzalkonium chloride were tested for their cytotoxicity against human keratinocytes (HaCaT cell line) in the MTT assay. Results are presented as IC_50_ (Table 1). BAC was determined to be highly cytotoxic (IC_50_ 1.1 ± 1.4 μg/mL). Only QAS **10** exhibited cytotoxic effect on HaCaT with IC_50_ 12.8 ± 1.2 μg/mL. QAS **10** proved to be 10 times less cytotoxic than commercially available benzalkonium chloride. QAS **3**–**9** did not cause 50% inhibition of the growth in the concentration range used in experiments.

### 2.3. Mutagenic Activity

**Figure 2:**
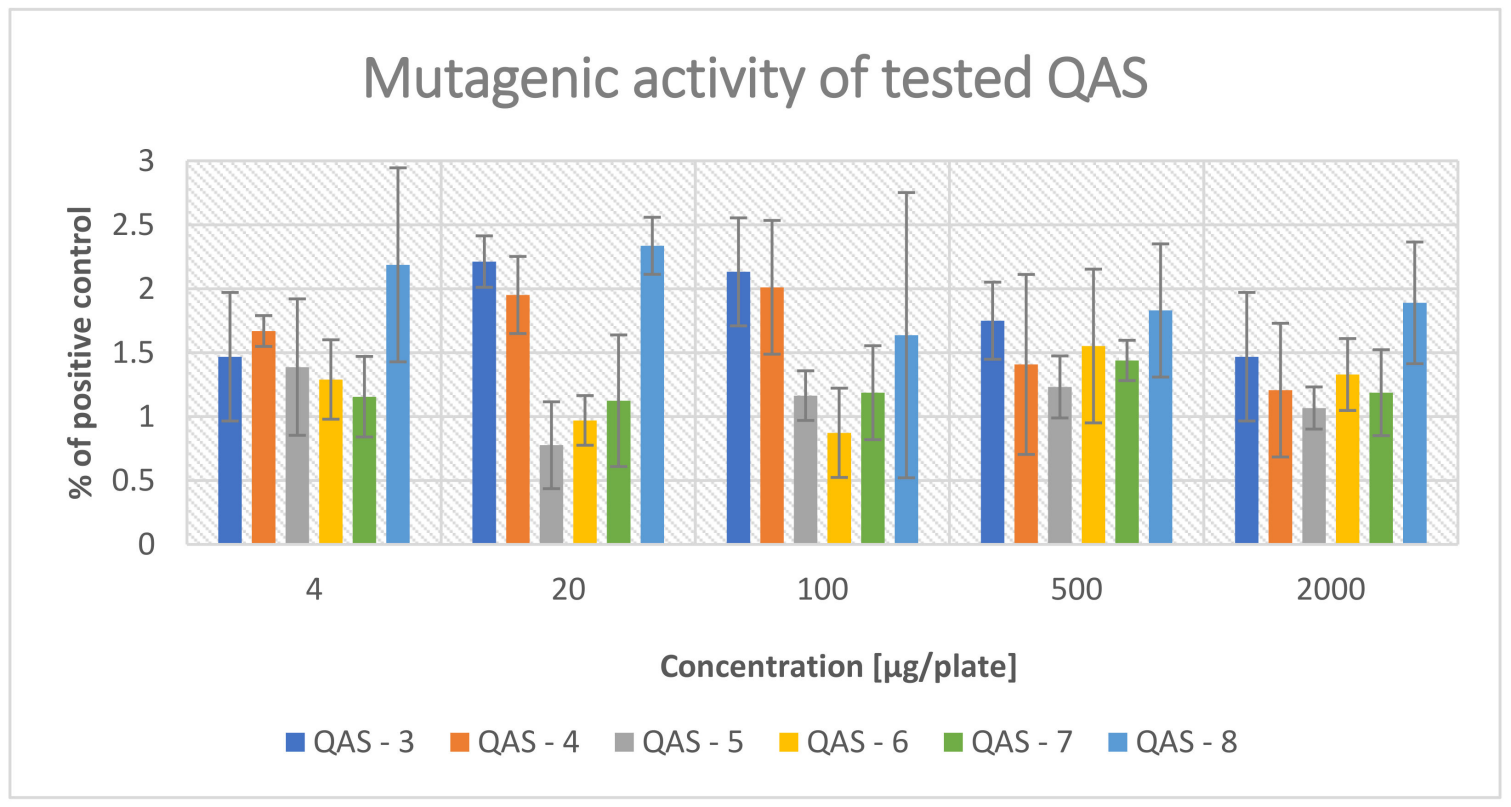
Mutagenic activity of QAS 3–8 presented as % of positive control (doxorubicin, 90 ng/plate) in Ames test with Salmonella typhimurium TA 98 strain.

Although QAS were generally considered to be safe chemicals, some reports suggest potential risks associated with their usage. Hence, those synthesized in this study were tested for mutagenicity using *Salmonella typhimurium* TA98 strain in the Ames test. In this test, histidine-dependent bacteria mutants are applied. Without histidine, no bacterial growth can be noticed, but each mutagenic substance induces a change in DNA and restores the ability to the biosynthesis of histidine in bacteria. This test is commonly used and was recommended by OECD to detect mutagenic potential of chemicals [50]. All synthesized QAS, except **9** and **10**, were submitted for testing in the Ames test. Salts **9** and **10** were excluded due to insufficient solubility. The results for QAS, both negative and positive controls, are presented in Table 3 and Figure 2. All tested QAS did not exhibit mutagenic activity over the concentration range of 4–2000 µg/plate. QAS activity is similar to that of a negative control representing spontaneous revertants and does not exceed 2.5% of the positive control, even at the highest concentrations. According to literature, BAC does not induce mutagenicity in Ames test in the concentration range 10–100 µg/plate [51].

## 3. Materials and Methods

### 3.1. Synthesis

#### 3.1.1. General Information

All reactions were monitored by thin-layer chromatography (TLC), using pre-coated silica gel 60 F_254_ plates (100–200 mesh). Spots were detected by spraying with a 5% solution of H_2_SO_4_ in methanol and charring. Purification of the products was performed using FLASH (PuriFlash 450, Interchim Montlucon Cedex, France) chromatography on pre-packed columns, 30 µm silica gel was a stationary phase.

The structures of all products were determined with the ^1^H, ^13^C, and 2D (HSQC and COSY) NMR, on Bruker AVANCE III (Bruker, Billerica, MA, USA), at 500 MHz and 125 MHz for ^1^H, ^13^C, respectively, in CDCl_3_, CD_3_OD, or D_2_O as solvents. High-resolution mass spectrometry analysis (HRMS) was performed using electrospray ionization with time-of-flight mass analysis (6550 iFunnel Q-TOF, Agilent Technologies, Santa Clara, CA, USA). Optical rotation was measured with a 343 PerkinElmer polarimeter (PerkinElmer, Waltham, MA, USA).

The purity of all QAS was confirmed by reversed-phase high-performance liquid chromatography (RP-HPLC). The equipment used was Varian Prostar 210 in high pressure gradient mode, Varian Prostar 325 UV (Varian Chroatography Systems, Walnut Creek, CA, USA) detector and Young Lin YL9181 ELSD detector (Young Lin Instrument CO, Anyang, Korea). All analyses were carried out on a Zorbax SB-C8 column (4.6 × 250 mm, 5 µm particle size) and UV detection at 254 nm for aromatic compounds and ELSD for aliphatic were used. Samples were eluted with a linear 1–90% acetonitrile gradient in deionized water over 20 min. The mobile phase flow rate was 1 mL/min. Both eluents contained 0.1% (*v/v*) of TFA. The purity of all QAS was >95%.

#### 3.1.2. *N*,*N*’-(1,4:3,6-Dianhydro-2,5-dideoxy-l-iditol-2,5-diyl)-bis[(3-carboxamide)pyridinium] Ditrifluoromethanesulfonate (**5**)

1,4:3,6-dianhydro-2,5-di-*O*-trifluoromethanesulfonyl-d-mannitol (**2**) (100 mg; 0.24 mmol) was placed in a screw-capped ampoule and dissolved in 1 mL of CH_3_CN, then 56.8 mg (0.46 mmol) of pyridine-3-carboxamide was added. The mixture was placed in a heating block, under nitrogen, at 70 °C for 5 days. Then, the solvent was evaporated. The product was purified by FLASH chromatography with DCM: MeOH gradient elution (100% DCM 0–10 min, 100−70% DCM 10–60 min, 70% DCM 60–120 min) using a 4 g silica gel prepacked column. Product **5**, a white solid, was obtained in the yield: 133 mg, (56%); mp: 63.5–65.2 °C, *R_f_* = 0 (acetone: hexane; 2:5). ${\left[\alpha \right]}_{D}^{20}$ = + 40.0° (*c* 0.3, MeOH). ^1^H NMR (500 MHz, D_2_O): 4.62 (1H, dd, *J*_1,1a_ = 12.3 Hz, H1′ (or H6_a_)); 4.66 (1H, dd, *J*_1a,2_ = 5.4 Hz, H1_a_ (or H6_a_)); 5.47 (1H, s, H3 (or H4)); 5.70 (1H, dd, *J*_1,2_ = 12.3 Hz, H2 (or H5)); 8.23 (1H, dd, aromatic); 8.94 (1H, d, *J* = 7.9 Hz, aromatic); 9.04 (1H, d, *J* = 6.6 Hz, aromatic) 9.26 (1H, s, aromatic). ^13^C NMR (125 MHz, D_2_O): 72.42 (C1 or C6); 75.86 (C2 or C5); 88.27 (C3 or C4); 119.58 (-CF_3_); 129.18 (aromatic); 134.57 (aromatic); 143.46 (aromatic); 145.19 (aromatic); 145.33 (aromatic); 165.40 (aromatic). HRMS (ESI-QTOF): *m/z* [M]^2+^ 178.0740 (C_18_H_20_N_4_O_4_^2+^); theoretical *m/z* [M]^2+^ 178.0737.

Copies of NMR and HRMS spectra—Figures S1–S5 in Supplementary Materials.

#### 3.1.3. *N,N’*-(1,4:3,6-Dianhydro-2,5-dideoxy-l-iditol-2,5-diyl)-bis(*N*-methylimidazolium) Ditrifluoromethanesulfonate (**6**)

100 mg (0.24 mmol) of **2** was placed in the screw capped ampoule and 0.1 mL of *N*-methylimidazole was added. The mixture was shaken for 24 h, under nitrogen at room temperature. Next, it was dissolved in 10 mL of water and extracted with chloroform (2 × 5 mL). After evaporation of water under reduced pressure product **6** was obtained as a white solid. Yield: 133 mg, 96%; mp: 90.5–93.7 °C; *R_f_* = 0 (acetone: hexane; 2:5). ${\left[\alpha \right]}_{D}^{20}$ = + 42.0° (*c* 0.3, MeOH). ^1^H NMR (500 MHz, D_2_O): 4.23 (3H, s, *N*-CH_3_ imidazole) 4.80 (2H, m, H1 and H1_a_ (or H6 and H6_a_)); 5.44 (1H, s, H3 (or H4)); 5.55 (1H, broad signal, H2 (or H5)); 7.83 (1H, s, imidazole ring); 7.85 (1H, s, imidazole ring). ^13^C NMR (125 MHz, D_2_O): 36.36 (*N*-CH_3_ imidazole); 65.13 (C2 or C5); 72.17 (C1 or C6); 87.44 (C3 or C4); 121.30 (imidazole ring); 124.94 (imidazole ring). HRMS (ESI-QTOF): *m*/*z* [M]^2+^ 138.0790 (C_14_H_20_N_4_O_2_^2+^); theoretical *m*/*z* [M]^2+^ 138.0788.

Copies of NMR and HRMS spectra—Figures S6–S10 in Supplementary Materials.

#### 3.1.4. *N*,*N*’-(1,4:3,6-Dianhydro-2,5-dideoxy-l-iditol-2,5-diyl)-bis(*N*,*N*-dimethyl-*N*-hexylammonium) Ditrifluoromethanesulfonate (**8**)

150 mg (0.37 mmol) of **2** was placed in a screw-capped ampoule and dissolved in 1 mL of CH_3_CN, then 0.165 mL (0.95 mmol) of *N,N*-dimethylhexylamine was added. The mixture was heated in the heating block, under a nitrogen atmosphere, at 70 °C for 24 h. Then time the solvent and the excess of amine were evaporated under reduced pressure in a rotary evaporator. Next, it was dissolved in methanol and activated carbon was added. After filtration, evaporation of the solvent, and crystallization from a methanol/water mixture product **8** was obtained as a white solid. Yield: 194 mg, 79%; mp: 222.8–224.0 °C; *R_f_* = 0 (acetone: hexane; 2:5). ${\left[\alpha \right]}_{D}^{20}$ = + 27.0° (*c* 0.3 MeOH). ^1^H NMR (500 MHz, CD_3_OD): 3.18 (3H, s, N(CH_3_)_a_); 3.20 (3H, s, N(CH_3_)_b_); 4.24 (1H, t, *J*_1a,2_ = *J*_1,2_ = 5.7 Hz H2 (or H5)); 4.35 (1H, dd, H1_a_ (or H6_a_)); 4.41 (1H, dd, *J*_1,1′_ = 11.4 Hz H1 (or H6)); 5.39 (1H, s, H3 (or H4)); Hexyl residue: 0.96 (3H, t, *J* = 6.9 Hz, CH_3_); 1.37 (6H, m, 3 × CH_2_); 1.85 (2H, m, CH_2_); 3.47 (2H, m, CH_2_). ^13^C NMR (125 MHz, CD_3_OD): 48.73 (N(CH_3_)_2_); 66.08 (C1 or C6); 76.67 (C2 or C5); 81.98 (C3 or C4); Hexyl residue: 12.79 (CH_3_), 21.95, 22.06, 25.51, 30.93 (4 × CH_2_); 65.16 (CH_2_). HRMS (ESI-QTOF): *m*/*z* [M]^2+^ 185.1776 (C_22_H_46_N_2_O_2_^2+^); theoretical *m*/*z* [M]^2+^ 185.1774.

Copies of NMR and HRMS spectra—Figures S11–S15 in Supplementary Materials.

#### 3.1.5. *N*,*N*’-(1,4:3,6-Dianhydro-2,5-dideoxy-l-iditol-2,5-diyl)-bis(*N*,*N*-dimethyl-*N*-decylammonium) Ditrifluoromethanesulfonate (**10**)

150 mg (0.37 mmol) of **2** was placed in the screw-capped ampoule and dissolved in 1 mL of CH_3_CN, then 0.237 mL (0.99 mmol) of *N,N*-dimethylhexylamine was added. The mixture was kept for 24 h in a heating block, under a nitrogen atmosphere, at 70 °C. Then, the solvent and the excess of amine were evaporated under reduced pressure in a rotary evaporator. Next, it was dissolved in methanol and activated carbon was added. After filtration, evaporation of the solvent, and crystallization from methanol/water mixture the product **10** was obtained as a white solid. Yield: 173 mg, 61%; mp: 191 °C (decomposition); *R_f_* = 0 (acetone: hexane; 2:5). ${\left[\alpha \right]}_{D}^{20}$ = + 19.0° (*c* 0.3 MeOH). ^1^H NMR (500 MHz, CD_3_OD): 3.18 (3H, s, N(CH_3_)_a_); 3.20 (3H, s, N(CH_3_)_b_); 4.27 (1H, t, *J*_1′,2_ = *J*_1,2_ = 5.9 Hz H2 (or H5)); 4.35 (1H, dd, H1_a_ (or H6 _a_)); 4.41 (1H, dd, *J*_1,1a_ = 11.4 Hz H1 (or H6)); 5.39 (1H, s, H3 (or H4)); Decyl residue: 0.92 (3H, t, *J* = 6.7 Hz, CH_3_); 1.34 (12H, m, 6 x CH_2_); 1.42 (2H, m); 1.86 (2H, m); 3.49 (2H, m, CH_2_); ^13^C NMR (125 MHz, CD_3_OD): 48.45 (N(CH_3_)_a_); 48.73 (N(CH_3_)_b_); 66.08 (C1 or C6); 76.68 (C2 or C5); 81.96 (C3 or C4); Decyl residue: 12.99 (CH_3_), 22.01, 22.30, 25.86, 28.79, 28.98, 29.11, 29.15, 31.62 (8 × CH_2_); 65.15 (CH_2_). HRMS (ESI-QTOF): *m*/*z* [M]^2+^ 241.2401 (C_30_H_62_N_2_O_2_^2+^); theoretical *m*/*z* [M]^2+^ 241.2400.

Copies of NMR and HRMS spectra—Figures S16–S20 in Supplementary Materials.

### 3.2. Antimicrobial Activity

The antimicrobial potential of the tested agents was evaluated against two reference strains of bacteria: *Staphylococcus aureus* ATCC 25923 and *Escherichia coli* K12, two reference strains of pathogenic yeasts, *Candida albicans* ATCC10231 and *Candida glabrata* DSMZ11226, as well as 20 strains of clinical isolates of *S. aureus* collected from patients with skin and soft tissue infections (*n* = 8) and strains derived from subclinical bovine mastitis milk samples (*n* = 12). Four of these strains, two isolated from human infections and two isolated from bovine mastitis, were classified as MRSA (Methicillin-resistant *Staphylococcus aureus*). All of them were *mecA* positive and their methicillin resistance was confirmed with the disc diffusion assay. The remaining 16 strains were MSSA (Methicillin-sensitive *Staphylococcus aureus*). The assays were performed using a serial, two-fold dilution method in 96-well microtiter plates under conditions recommended by the Clinical and Laboratory Standards Institute (CLSI, Pittsburgh, PA, USA). The aim of this procedure was the determination of the MIC parameter (Minimum Inhibitory Concentration)—the minimum concentration of a tested agent capable to inhibit the growth of a specified strain of microorganism. The agents were solubilized in deionized, sterile water to a final concentration of 16 mg/mL (16,000 µg/mL). Subsequently, solutions were mixed (1:1 (*v/v*)) with 2 × concentrated liquid medium, Mueller-Hinton Broth 2—cation adjusted (MHB2) for evaluation of antibacterial activity and RPMI in the case of evaluation of the antifungal activity. QAS with lower solubility in water (**8**, **9** and **10**) were first dissolved in DMSO up to a final concentration of 100 mg/mL (100,000 µg/mL). Subsequently, the solutions were mixed with MHB2 or RPMI medium to get the final concentration of 8.192 mg/mL (8192 µg/mL). In the next step, the serial, two-fold dilutions of the tested agents (over the range of concentrations from 8192 to 8 µg/mL for reference strains of bacteria and yeasts and from 512 to 1.0 µg/mL for clinical isolates of *S. aureus*) were prepared in 96-well microtitration plates in the final volume of 100 μL of the appropriate medium.

The pure bacterial cultures of (both the reference and clinical strains) were grown on the Mueller-Hinton Agar (MHA) for 18–24 h at 37 °C. A small amount of the biomass of the culture of each strain of microorganisms was suspended in the sterile PBS (phosphate buffered saline, pH 7.4 at 25 °C, purchased from Sigma) solution to get an optical density OD_600_ = 0.13 (equal to the cells concentration approximately 1 × 10^8^ CFU/mL). The obtained suspensions of the cells were next diluted 1:100 (*v/v*) in the MHB2 medium. One hundred µL of the cells’ suspension was finally loaded into the wells of plates prepared in advance, which contained 100 µL of two-fold dilutions of the tested agents (the final concentration of the bacterial cells in all wells was approximately 5 × 10^5^ CFU/mL).

The pure cultures of yeast strains were grown on the Sabouraud Dextrose Agar (SDA) for 18–24 h at 37 °C. Suspensions of the microorganisms were prepared by taking one loop of pure culture into sterile water and adjusting optical density to 0.1 at 660 nm and further 50-fold dilution in RPMI 1640 medium resulting in cells concentration of approximate 2 × 10^4^ CFU/mL. One hundred μL of that suspension was inoculated to the wells of the microtitration plate containing appropriate dilutions of the agents. The positive growth control of each strain (both bacteria and yeasts) was performed in the wells without the tested substances. The negative control containing only the media was included in each assay. Microtiter plates were incubated at 37 °C for 24 h. Following the incubation period, determination of the MIC values of the tested agents was carried out by measuring the absorbance at 531 nm using a Victor^3^ microplate reader (Perkin Elmer, Inc., Waltham, MA, USA). The lowest concentration of the agent causing inhibition of growth equal to or higher than 90% (MIC_90_) of growth control was taken as the MIC value. Each test was repeated three times.

### 3.3. Toxicity—MTT Assay

To assess the cytotoxicity of QAS (IC_50_), the MTT assay on 96-well polystyrene plates was performed for human keratinocytes (HaCaT, Elabscience Biotechnology Inc., Houston, Texas, USA). The assay utilizes colorimetric determination of the cell metabolic activity and the color intensity reflects the number of live cells that can be measured spectrophotometrically. The cell line was cultured in a Dulbecco’s modified Eagle Medium (Invitrogen) supplemented with 10% fetal bovine serum (Sigma Aldrich) (*v/v*), 100 units/mL of penicillin, 100 μg/mL of streptomycin, and 2 mM l-glutamine and was stored at 37 °C in a humidified 5% CO_2_ incubator. Briefly, a day after plating of 500 cells per well, a series of concentrations (0.5–200 μg/mL) of the test compounds was applied. DMSO was added to the control cells at a final concentration of 1.0% (*v/v*), which was related to the maximal concentration of the solvent compounds used in the experiment. After 24 h of incubation with the QAS at 37 °C (humidified 5% CO_2_ incubator), a medium containing 1 mg/mL of MTT (3-(4,5-dimethylthiazol-2-yl)-2,5-diphenyltetrazolium bromide) was added to the wells up to a final concentration of 0.5 mg/mL. Subsequently, the plates were incubated at 37 °C for 4 h. Then, the medium was removed by suction, and the formazan product was solubilized with DMSO. The background absorbance at 630 nm was subtracted from that at 570 nm for each well (Epoch, BioTek Instruments, Winooski, VT, USA). Six replicates were conducted for each concentration. All experiments were repeated at least twice and the resulting IC50 values were calculated with GraFit 7 software (v. 7.0, Erithacus, Berkley, CA, USA).

### 3.4. Mutagenic Assay—The Ames Test

A Salmonella mutagenicity test was performed with *Salmonella typhimurium* TA98 strain (Xenometrics AG, Allschwil, Switzerland) without metabolic activation, following the procedure by Mortelmans and Zeiger [52], with modifications reported Golunski et al. [53] A mixture containing 100 µL of the overnight culture of *S. typhimurium* TA98, 50 µL of 3% NaCl, and 100 µL of the test chemical dilution (or sterile distilled water as a negative control) was incubated for 4 h in darkness at 37 °C and 220 rpm. Subsequently, the mixture was centrifuged, bacterial pellet washed with 0.75% NaCl and resuspended in 300 µL of 0.75% NaCl solution containing 0.1 µmol each of histidine and biotin, and it was then spread on a glucose minimal (GM) plate. The number of revertant colonies was counted after a 48-h incubation at 37 °C in the dark. All experiments were performed in triplicate. The anticancer drug doxorubicin at 90 ng/plate with known mutagenic effect towards *S. typhimurium* TA98 was used as a positive control. All tested compounds were nontoxic at concentrations up to 2 mg/plate, as determined by observation of the background lawn alterations. Mutagenic activity of two compounds, **9** and **10**, was not assessed due to their insufficient solubility in both water and DMSO. Ampicillin, histidine, biotin, and doxorubicin used in the Ames test were purchased from Sigma–Aldrich Chemical Company.

## 4. Conclusions

A series of gemini quaternary ammonium salts, derivatives of 1,4:3,6-dianhydro-l-iditol and aliphatic and aromatic amines, were synthesized. All QAS were tested for their antimicrobial activity against the yeast (*C. albicans* and *C. glabrata*) and bacterial (*E. coli* and *S. aureus*) reference strains. Two salts (**9** and **10**) exhibited antimicrobial activity, and the most active was compound **10**, containing decyl residues attached to quaternary nitrogen atoms with MIC values of 32, 16, 8, and 32 μg/mL for *C. albicans, C. glabrata, S. aureus,* and *E. coli,* respectively. Moreover, both compounds were tested for their activity against *S. aureus* clinical isolates. The activities of **9** and **10** were comparable to those against the reference strain, with a markedly higher for compound **10**. Only QAS **10** exhibited cytotoxic effect in MTT assay against human keratinocytes (HaCaT) with IC_50_ 12.8 ± 1.2 μg/mL. QAS **10** was less active against bacteria and yeast in comparison to that of benzalkonium chloride. However, **10** proved to be less toxic, as IC_50_ for BAC is about 10 times lower than for **10**. Furthermore, QAS were tested for their mutagenic potential in the Ames test. No tested substance showed mutagenic activity over the 4–2000 µg/plate concentration range. Gemini salts, derivatives of 1,4,3,6-dianhydro-l-iditol, were prepared in two steps from renewable and accessible material (isomannide) and proved to be nonmutagenic, while they proved active against a spectrum of bacteria and yeast. QAS derived from renewable resources, i.e., carbohydrates and alditols, turned out to be promising candidates to replace currently used QAS in laboratories, industry, agriculture, and medicine.

## Figures and Tables

**Table 1:** Antimicrobial activity-MIC ([**μg/mL**]/[*μM]*) and cytotoxicity-IC50 [μg/mL] for QAS **3**–**10**.

QAS	*C. albicans* ATCC 10231	*C. glabrata* DSMZ 11226	*S. aureus* ATCC 25923	*E. coli* K12	HaCaT IC_50_
3	>4096	>4096	>4096	>4096	>100
4	>4096	>4096	>4096	>4096	>100
5	>4096	>4096	>4096	>4096	>100
6	>4096	>4096	>4096	>4096	>100
7	>4096	>4096	>4096	>4096	>100
8	4000	2048	2048	>4096	>100
9	**1024**/*1408*	**1024**/*1408*	**128**/*176*	**2048**/*2816*	>100
10	**32**/*40*	**16**/*20*	**8**/*10*	**32**/*40*	12.8 ± 1.25

**Table 2:** MIC ([**μg/mL**]/[*μM]*) values for QAS **9** and **10** against *S. aureus* clinical isolates.

Number of *S. aureus* Isolate	QAS	
	9	10
1	**64**/*88*	**8**/*10*
2	**64**/*88*	**8**/*10*
3	**64**/*88*	**4**/*5*
4	**128**/*176*	**8**/*10*
5	**64**/*88*	**8**/*10*
6	**64**/*88*	**8**/*10*
7 *	**64**/*88*	**4**/*5*
8 *	**64**/*88*	**8**/*10*
9	**64**/*88*	**8**/*10*
10	**64**/*88*	**8**/*10*
11	**64**/*88*	**8**/*10*
12	**64**/*88*	**8**/*10*
13	**64**/*88*	**8**/*10*
14	**64**/*88*	**4**/*5*
15	**64**/*88*	**8**/*10*
16	**64**/*88*	**8**/*10*
17	**64**/*88*	**8**/*10*
18	**64**/*88*	**8**/*10*
19 *	**64**/*88*	**8**/*10*
20 *	**64**/*88*	**8**/*10*

Strains 1–8 were isolated from human infections; strains 9–20 were isolated from bovine mastitis; * MRSA strains.

**Table 3:** Mutagenic activity of QAS **3–10** in Ames test with *Salmonella typhimurium* TA 98 strain.

	Number of Revertants						
	Concentration [µg/plate]						
	4	20	100	500	2000	Positive Control Doxorubicin, 90 ng/Plate	Negative Control Water
**3**	7.3 ± 2.5	11.0 ± 1.0	10.6 ± 2.1	8.7 ± 1.5	7.3 ± 2.5	497.3 ± 19.8	10.3 ± 4.5
**4**	8.3 ± 0.6	9.7 ± 1.5	10.0 ± 2.6	7.0 ± 3.5	6.0 ± 2.6	497.3 ± 19.8	10.3 ± 4.5
**5**	14.3 ± 5.5	8.0 ± 3.5	12.0 ± 2.0	12.7 ± 2.5	11.0 ± 1.7	1031.2 ± 43.1	16.0 ± 1.0
**6**	13.3 ± 3.2	10.0 ± 2.0	9.0 ± 3.6	16.0 ± 6.2	13.7 ± 2.9	1031.2 ± 43.1	16.0 ± 1.0
**7**	11.0 ± 3.0	10.7 ± 4.9	11.3 ± 3.5	13.7 ± 1.5	11.3 ± 3.2	952.3 ± 120.3	17.3 ± 6.8
**8**	14.7 ± 5.1	15.7 ± 1.5	11.0 ± 7.5	12.3 ± 3.5	12.7 ± 3.2	672.3 ± 34.4	14.3 ± 1.9
**9**	Not determined-insoluble						
**10**	Not determined-insoluble						

## Data Availability

Not applicable.

## Supplementary Materials

The following are available online. Figure S1: HRMS (ESI-QTOF) of compound **5**, Figure S2: ^1^H NMR (500 MHz, D_2_O) of compound **5**, Figure S3: ^13^C NMR (125 MHz, D_2_O) of compound **5**, Figure S4: COSY of compound **5**, Figure S5: HSQC of compound **5**, Figure S6: HRMS (ESI-QTOF) of compound **6**, Figure S7: ^1^H NMR (500 MHz, D_2_O) of compound **6**, Figure S8: ^13^C NMR (125 MHz, D_2_O) of compound **6**, Figure S9: COSY of compound **6**, Figure S10: HSQC of compound **6**, Figure S11: HRMS (ESI-QTOF) of compound **8**, Figure S12: ^1^H NMR (500 MHz, CD_3_OD) of compound **8**, Figure S13: ^13^C NMR (125 MHz, CD_3_OD) of compound **8**, Figure S14: COSY of compound **8**, Figure S15: HSQC of compound **8**, Figure S16: HRMS (ESI-QTOF) of compound **10**, Figure S17: ^1^H NMR (500 MHz, CD_3_OD) of compound **10**, Figure S18: ^13^C NMR (125 MHz, CD_3_OD) of compound **10**, Figure S19: COSY of compound **10**, Figure S20: HSQC of compound **10**.
