# Supplementary material for: Antimicrobial, Cytotoxic and Mutagenic Activity of Gemini QAS Derivatives of 1,4:3,6-Dianhydro-l-iditol
Source: Molecules. 2022 Jan 24;27(3):757. doi: 10.3390/molecules27030757 (PMC8838521; doi:10.3390/molecules27030757)

Supplementary materials

Antimicrobial, Cytotoxic and Mutagenic Activity of Gemini QAS Derivatives of 1,4:3,6-Dianhydro-L-iditol

Karol Sikora [1,2,*], Andrzej Nowacki [2], Piotr Szweda [3], Anna Woziwodzka [4], Sylwia Bartoszewska [1], Jacek Piosik [4] and Barbara Dmochowska [2]

[1] Department of Inorganic Chemistry, Faculty of Pharmacy, Medical University of Gdańsk, Al. Gen. J. Hallera 107, 80-416 Gdańsk, Poland; sylwia.bartoszewska@gumed.edu.pl (S.B.)
[2] Faculty of Chemistry, University of Gdańsk, Wita Stwosza 63, 80-308 Gdańsk, Poland; andrzej.nowacki@ug.edu.pl (A.N.); basia.dmochowska@ug.edu.pl (B.D.)
[3] Department of Pharmaceutical Technology and Biochemistry, Faculty of Chemistry, Gdańsk University of Technology, ul. G. Narutowicza 11/12, 80-233 Gdańsk, Poland; piotr.szweda@pg.edu.pl
[4] Laboratory of Biophysics, Intercollegiate Faculty of Biotechnology, University of Gdańsk and Medical University of Gdańsk, Abrahama 58, 80-307 Gdańsk, Poland; anna.woziwodzka@biotech.ug.edu.pl (A.W.); jacek.piosik@biotech.ug.edu.pl (J.P.)
* Correspondence: karol.sikora@gumed.edu.pl

HRMS and NMR spectra of synthesised compounds

1. *N,N'*-(1,4:3,6-Dianhydro-2,5-dideoxy-L-iditol-2,5-diyl)-bis[(3-carboxamide)pyridinium] ditrifluoromethanesulfonate (5)

Figure S1: HRMS (ESI-QTOF) of compound **5**.

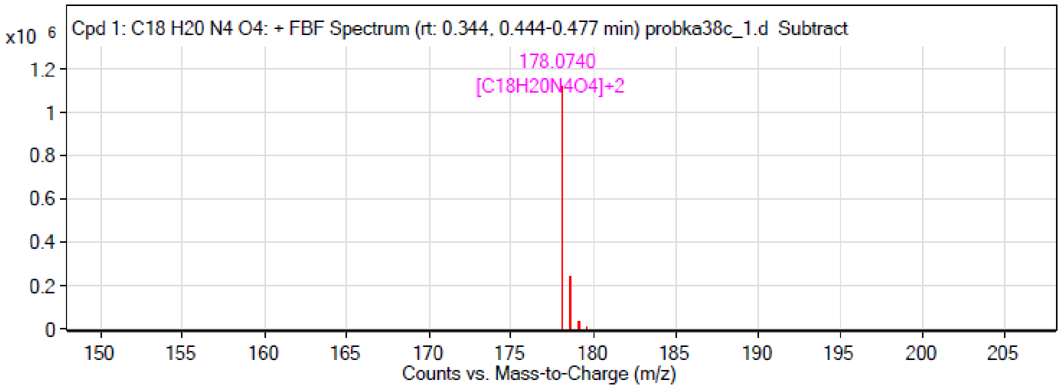

Figure S2: ^{1}H NMR (500 MHz, D_2O) of compound **5**.

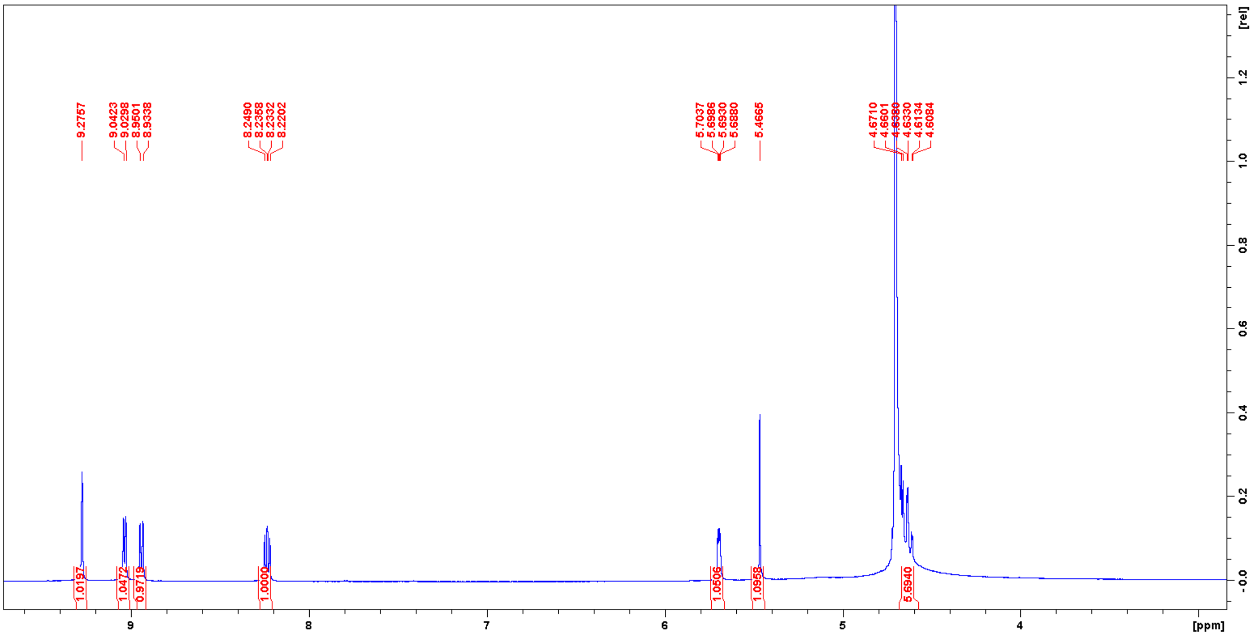

Figure S3: ^{13}C NMR (125 MHz, D_2O) of compound **5**.

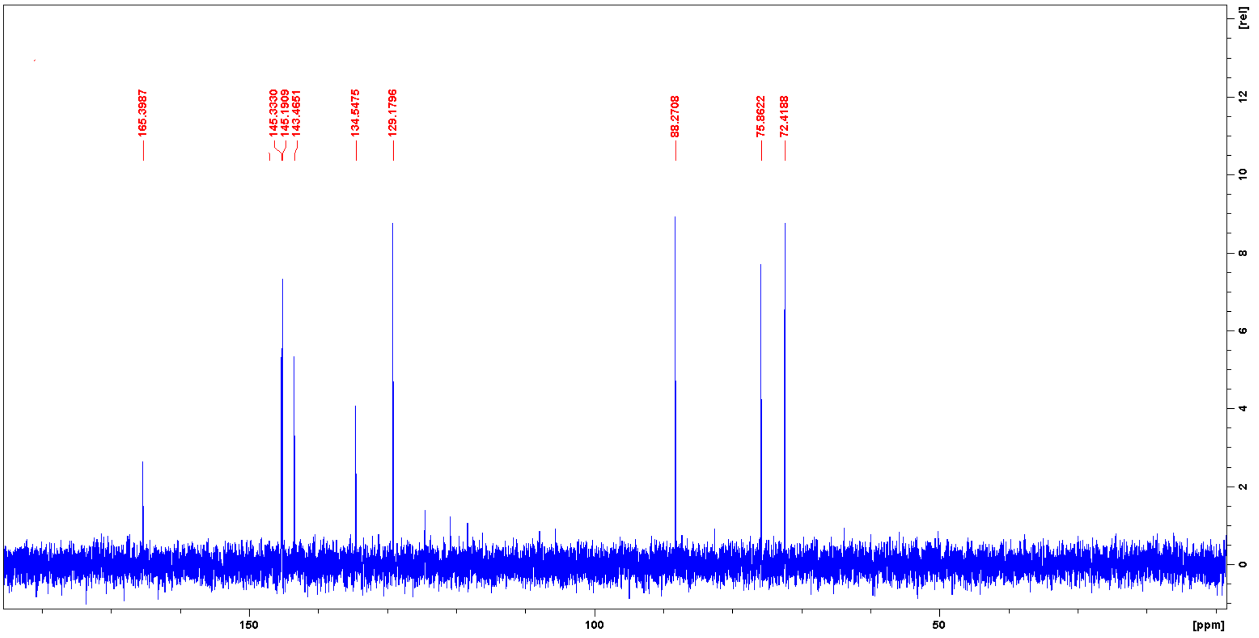

Figure S4: COSY of compound **5**.

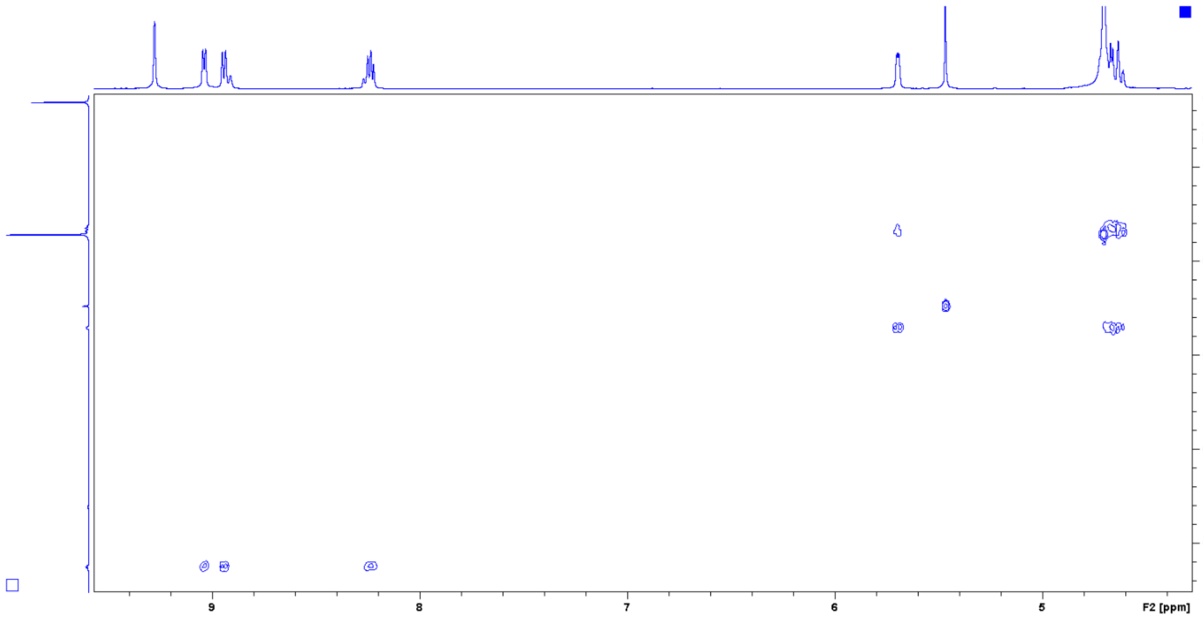

Figure S5: HSQC of compound **5**.

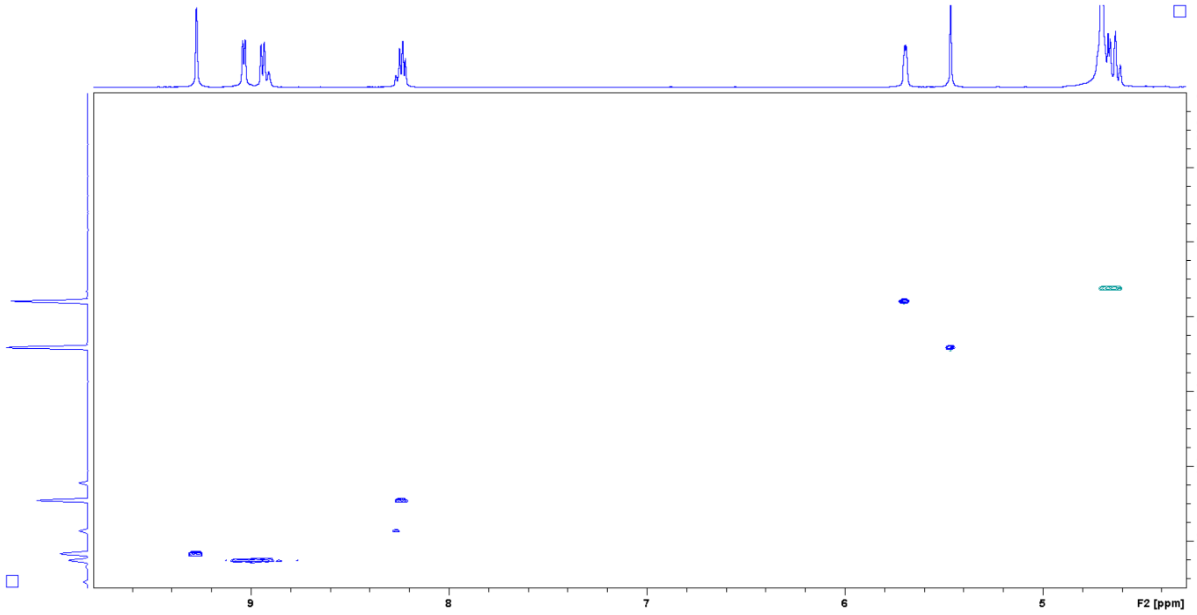

2. *N,N'*-(1,4:3,6-Dianhydro-2,5-dideoxy-L-iditol-2,5-diyl)-bis(*N*-methylimidazolium) ditrifluoromethanesulfonate (6)

Figure S6: HRMS (ESI-QTOF) of compound **6**.

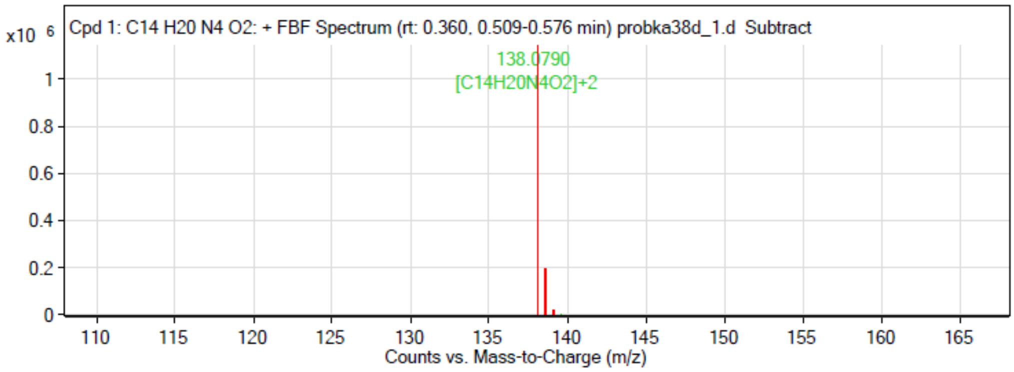

Figure S7: ^{1}H NMR (500 MHz, D_2O) of compound **6**.

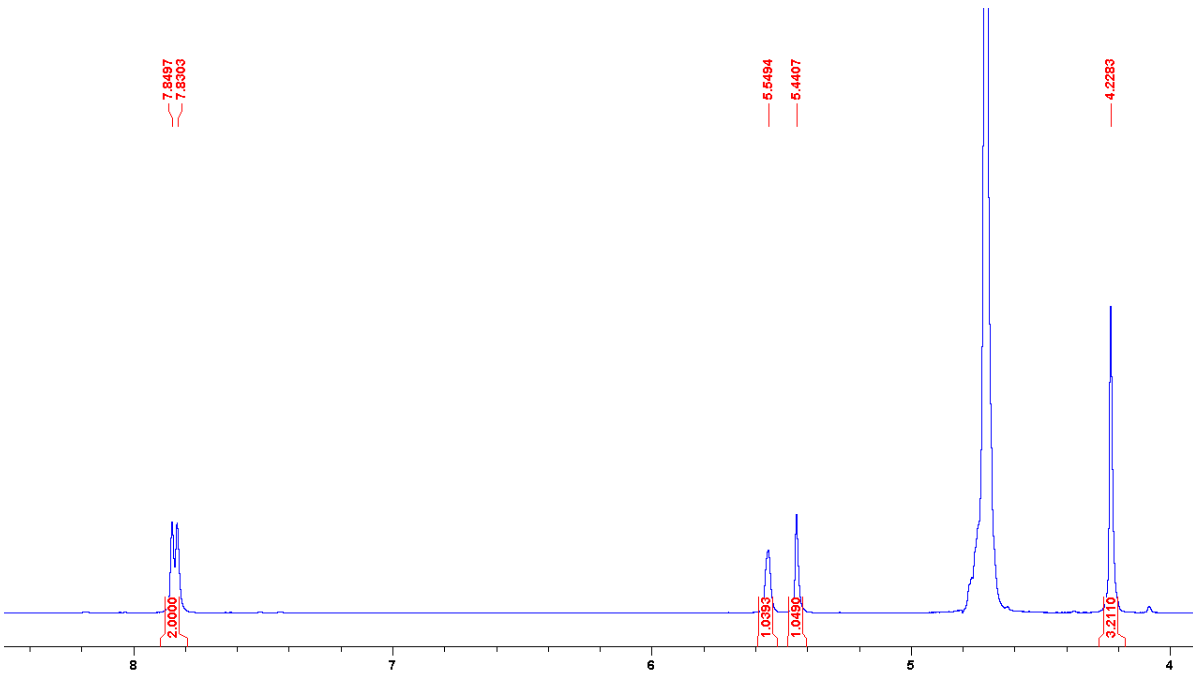

Figure S8: ^{13}C NMR (125 MHz, D_2O) of compound **6**.

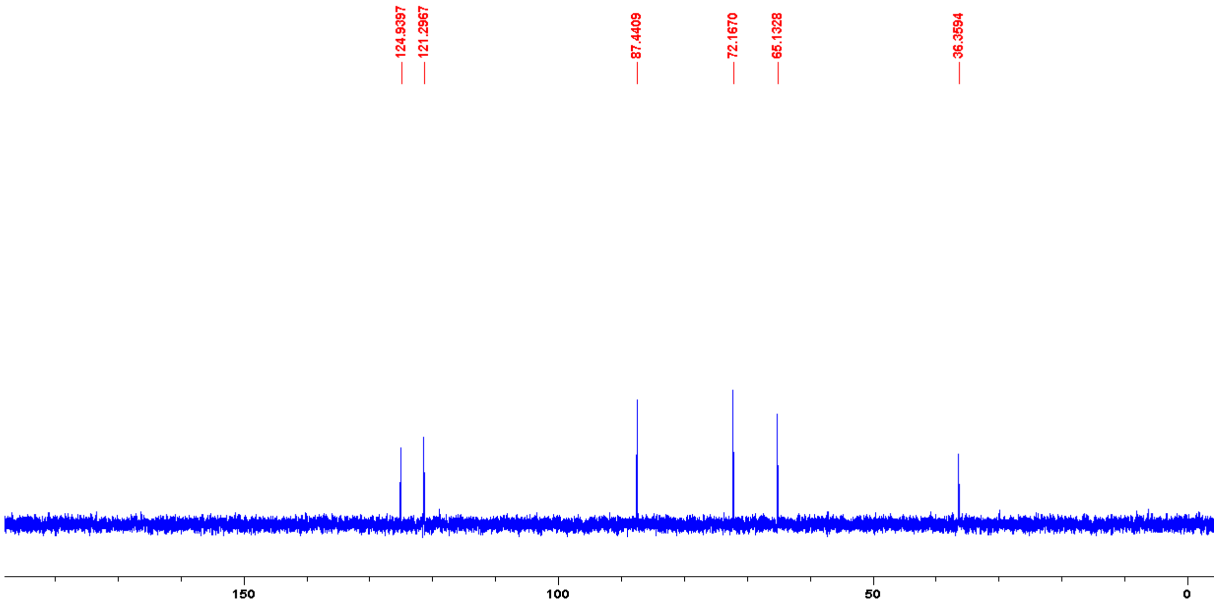

Figure S9: COSY of compound **6**.

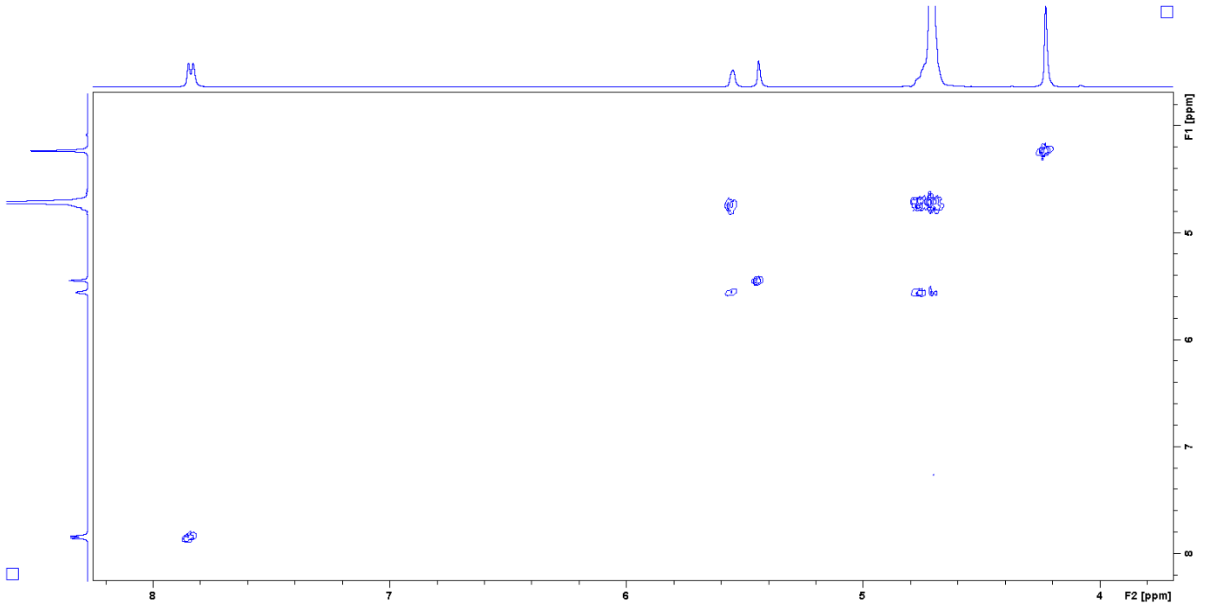

Figure S10: HSQC of compound **6**.

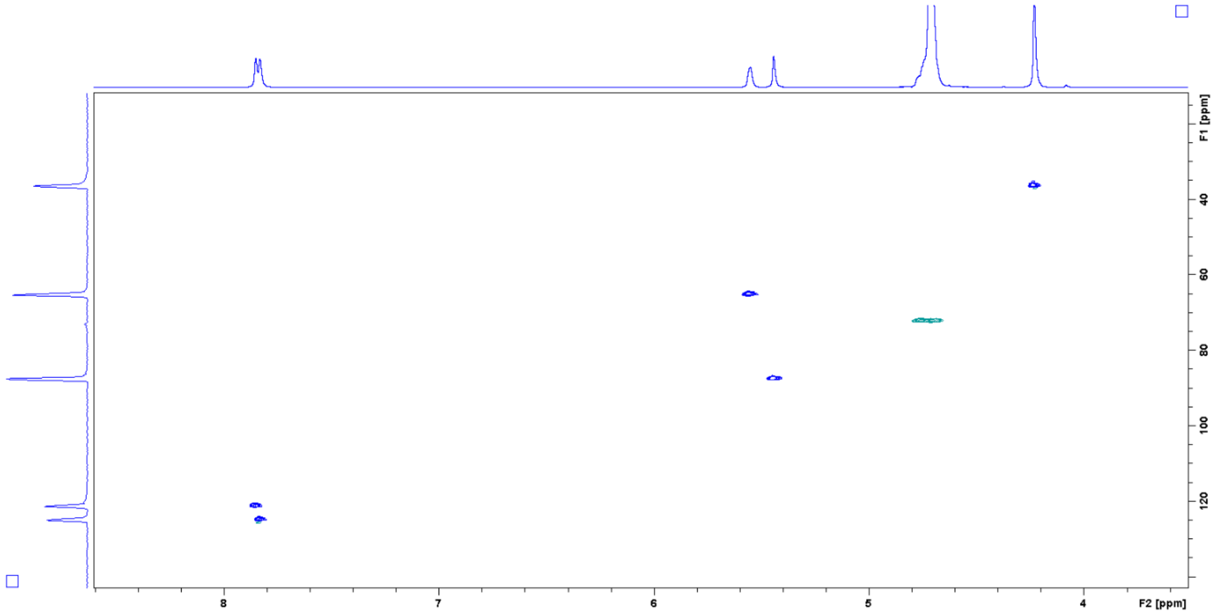

3. ***N*,*N'*-(1,4:3,6-Dianhydro-2,5-dideoxy-L-iditol-2,5-diyl)-bis(*N*,*N*-dimethyl-*N*-hexylammonium) ditrifluoromethanesulfonate (8)**

Figure S11: HRMS (ESI-QTOF) of compound **8**.

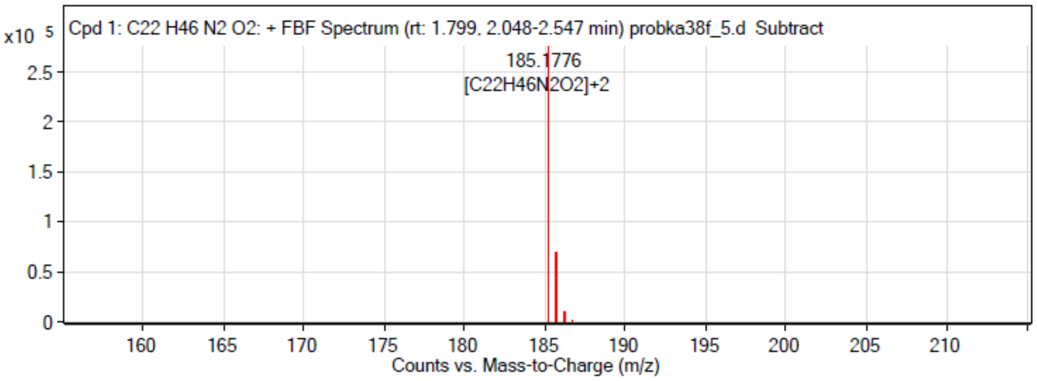

Figure S12: ^{1}H NMR (500 MHz, CD_3OD) of compound **8**.

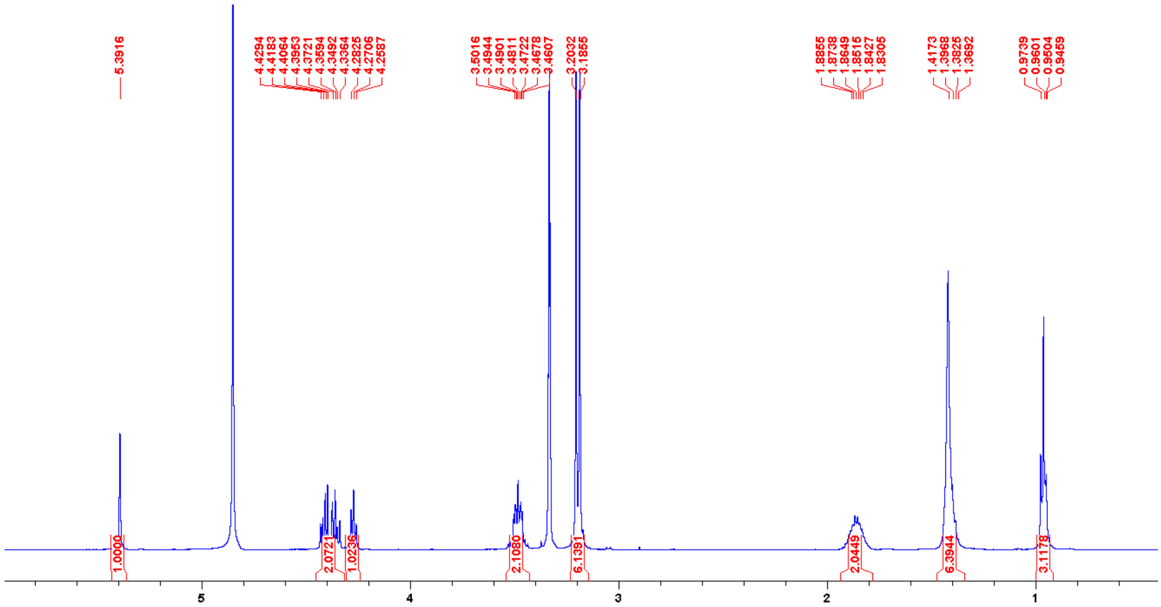

Figure S13: ^{13}C NMR (125 MHz, CD_3OD) of compound **8**.

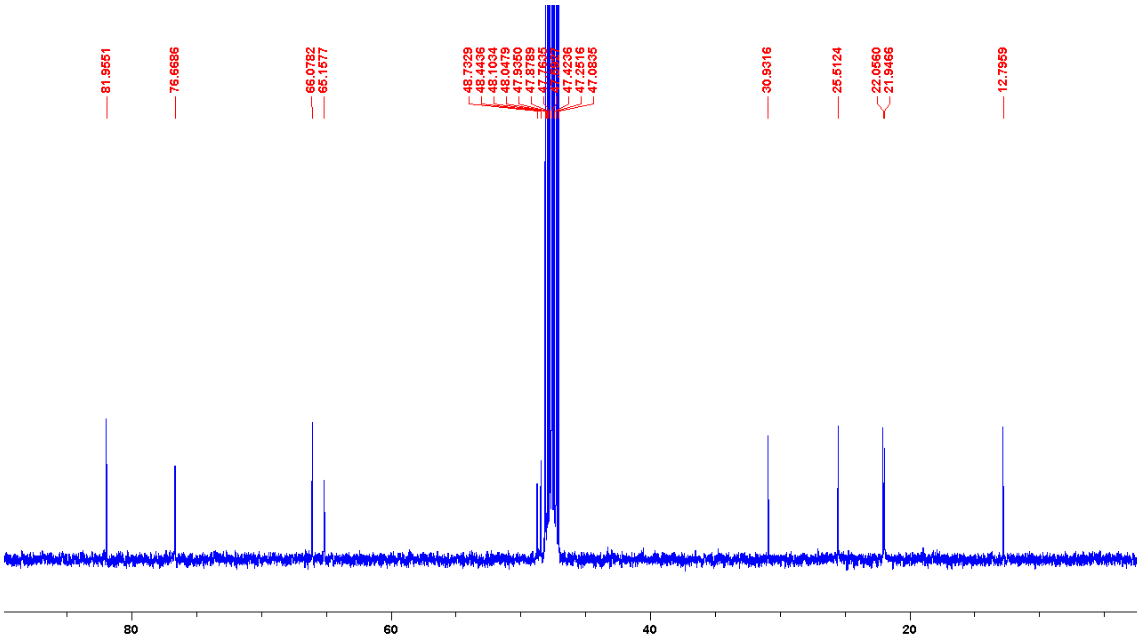

Figure S14: COSY of compound **8**.

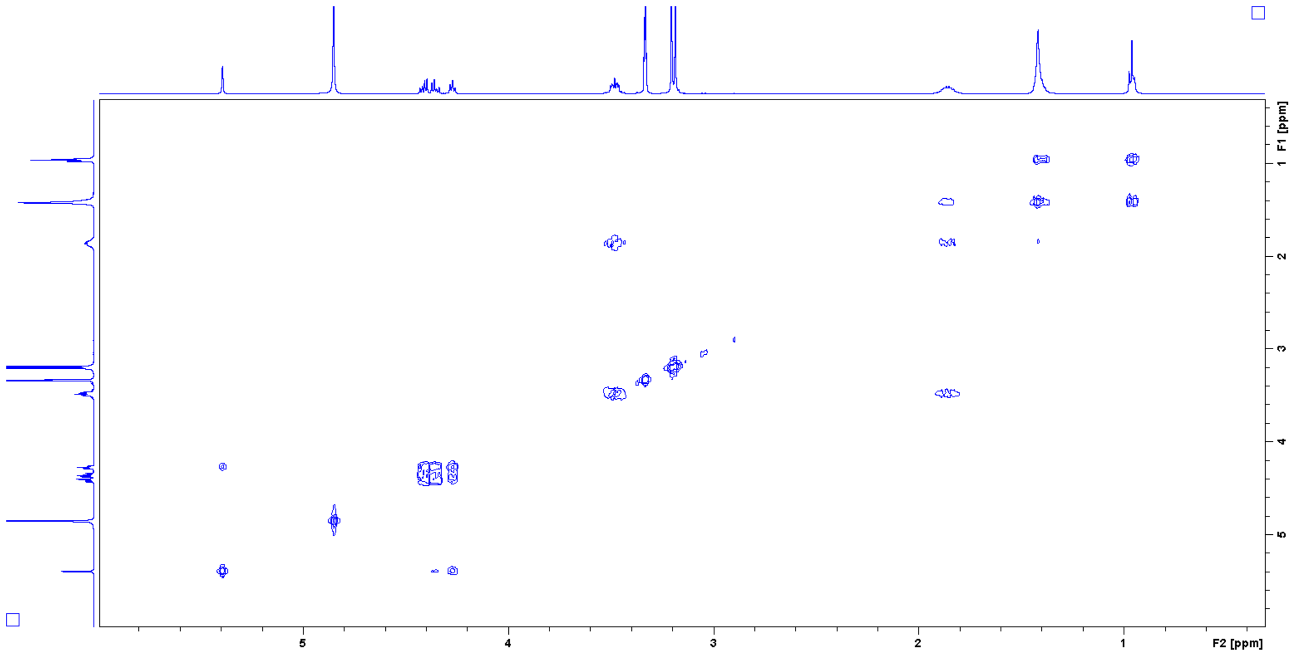

Figure S15: HSQC of compound **8**.

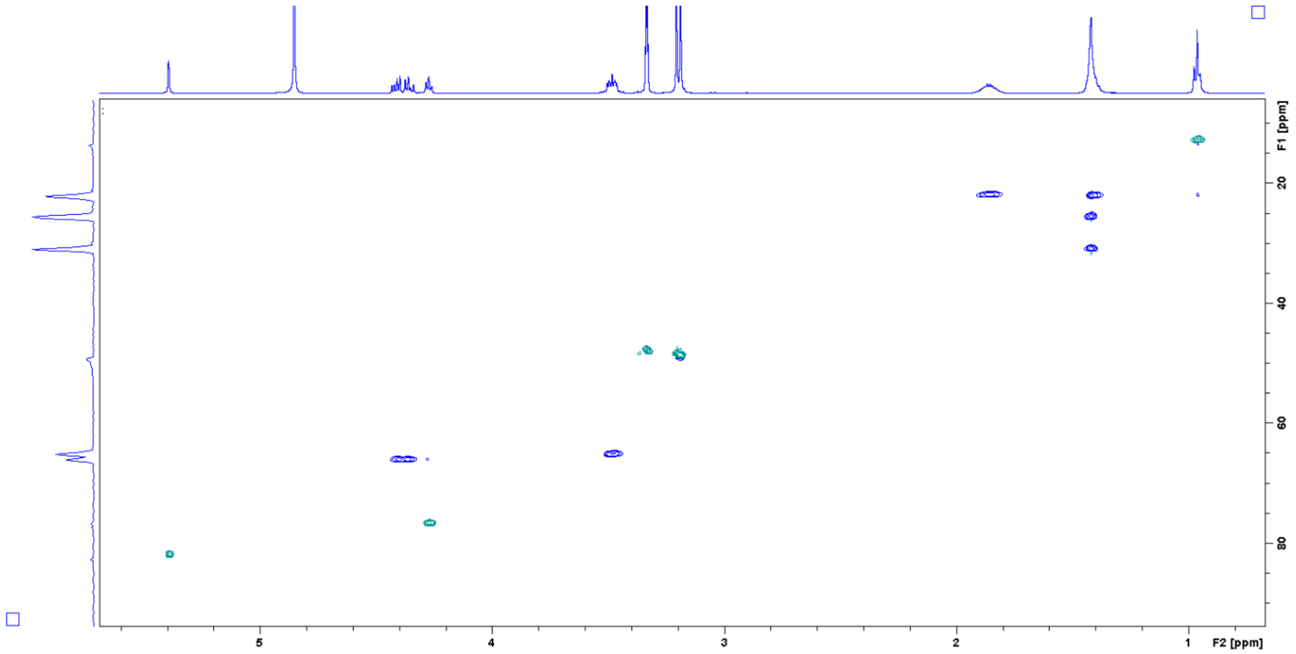

4. ***N,N'*-(1,4:3,6-Dianhydro-2,5-dideoxy-L-iditol-2,5-diyl)-bis(*N,N*-dimethyl-*N*-decylammonium) ditrifluoromethanesulfonate (10)**

Figure S16: HRMS (ESI-QTOF) of compound **10.**

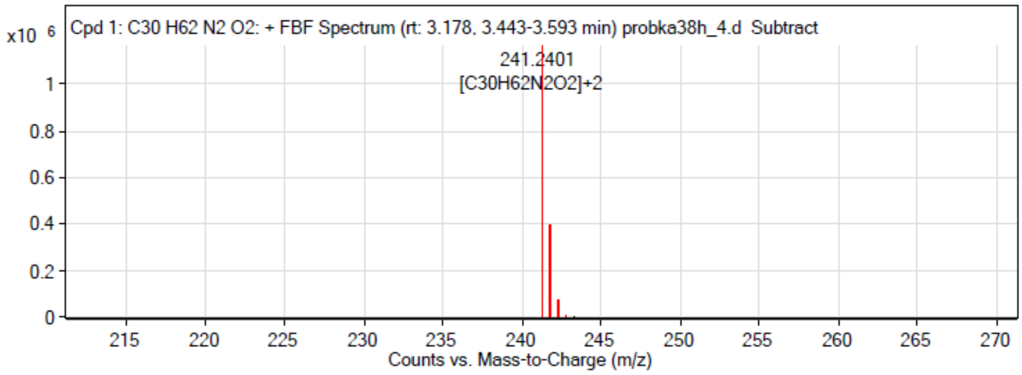

Figure S17: ^{1}H NMR (500 MHz, CD_3OD) of compound **10.**

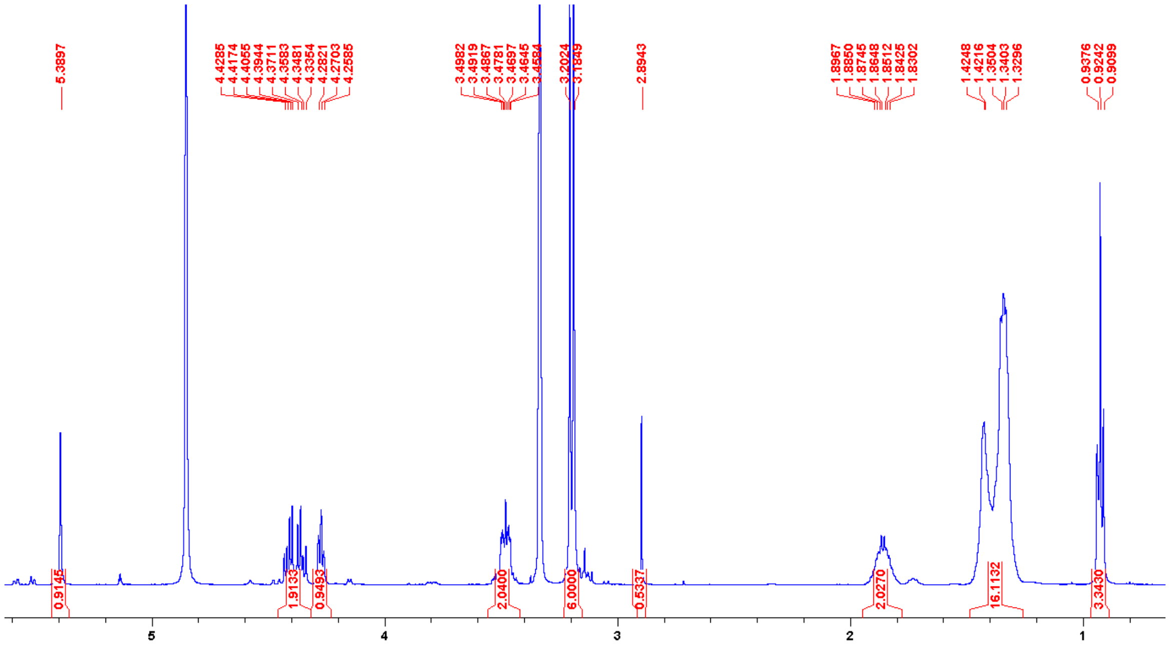

Figure S18: ^{13}C NMR (125 MHz, CD_3OD) of compound **10.**

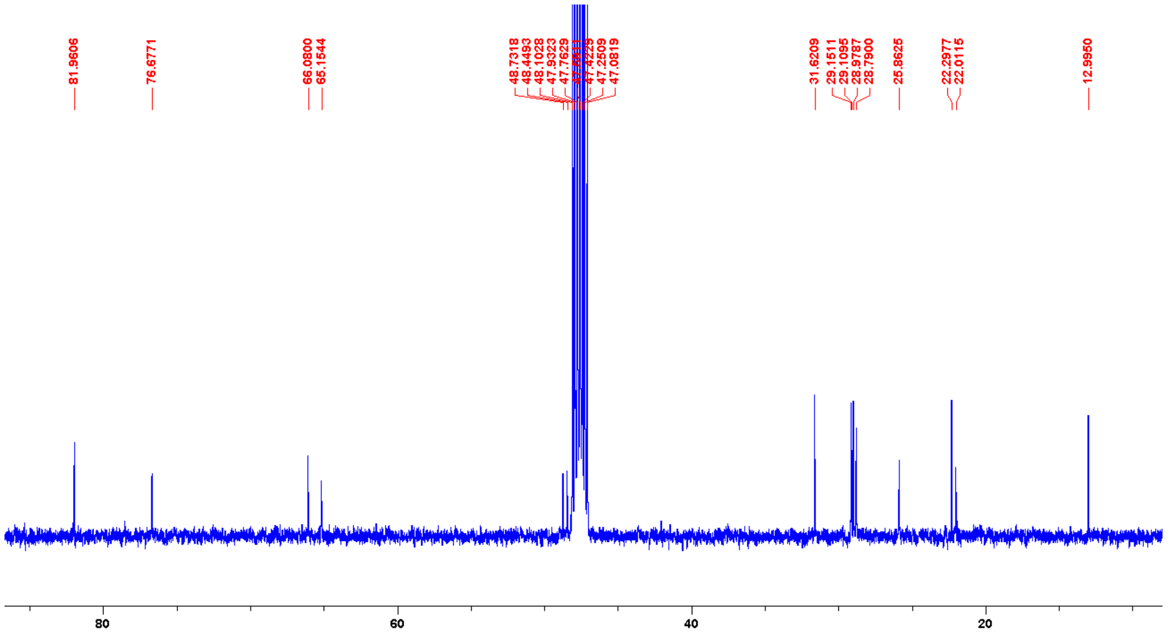

Figure S19: COSY of compound **10.**

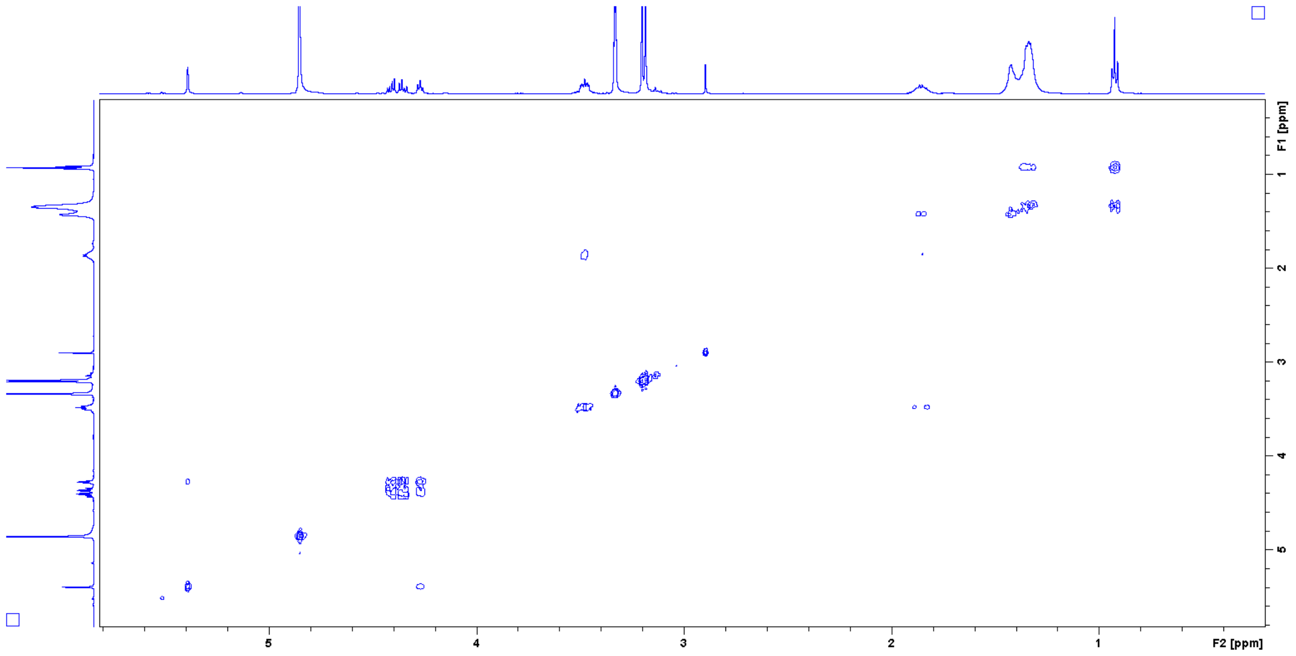

Figure S20: HSQC of compound **10.**

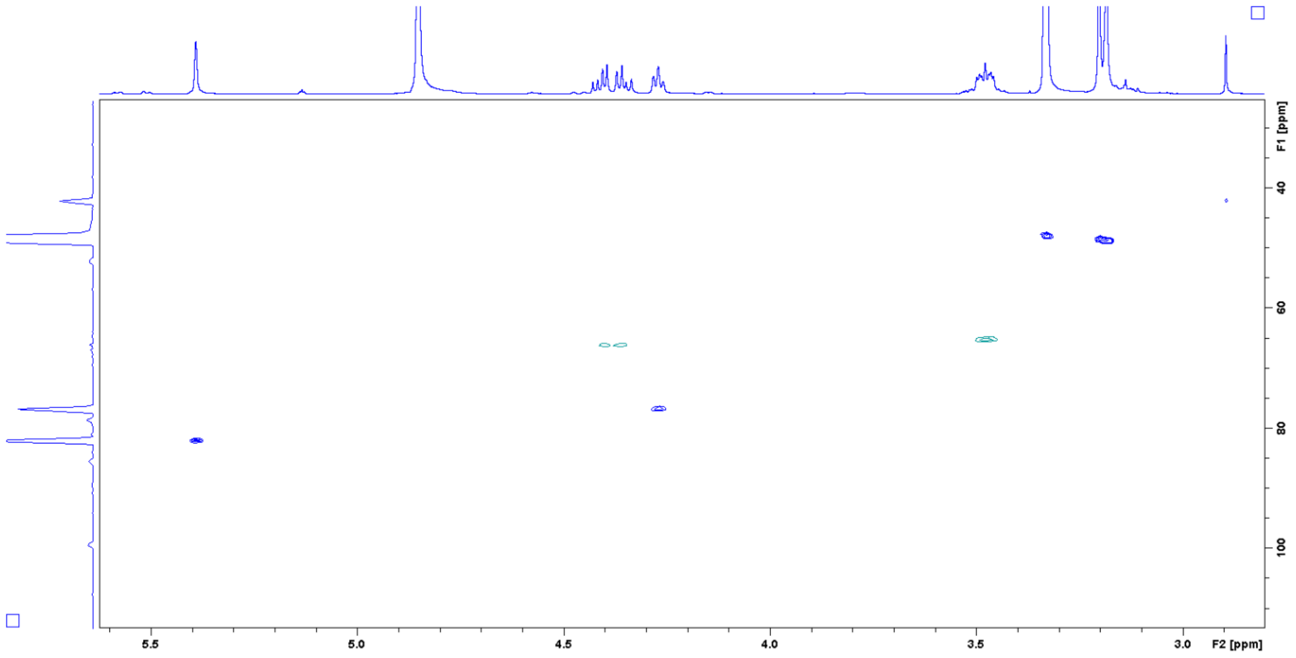

Supplement: Supplementary file 1 [file molecules-27-00757-s001.zip › molecules-1494662-supplementary.pdf]
